# *Bacillus subtilis* Inoculation Improves Nutrient Uptake and Physiological Activity in Sugarcane under Drought Stress

**DOI:** 10.3390/microorganisms10040809

**Published:** 2022-04-13

**Authors:** Mariley de Cássia da Fonseca, João William Bossolani, Sirlene Lopes de Oliveira, Luiz Gustavo Moretti, José Roberto Portugal, Daniele Scudeletti, Elisa Fidêncio de Oliveira, Carlos Alexandre Costa Crusciol

**Affiliations:** Department of Crop Science, College of Agricultural Sciences, São Paulo State University (UNESP), Botucatu 18610-034, Sao Paulo, Brazil; marileycfonseca@gmail.com (M.d.C.d.F.); bossolani.agro@gmail.com (J.W.B.); sirlene.lopes@unesp.br (S.L.d.O.); souzamoretti@gmail.com (L.G.M.); jose.portugal@unesp.br (J.R.P.); daniele.scudeletti@hotmail.com (D.S.); elisa_oliveiraagro@hotmail.com (E.F.d.O.)

**Keywords:** water restriction, plant growth-promoting bacteria, net photosynthetic rate, antioxidant enzymes

## Abstract

Sugarcane (*Saccharum* spp.) is one of the most important crops in the world. Throughout the sugarcane’s growth stages, periods of drought are common, causing detrimental effects on plant growth. Therefore, the search for strategies for minimizing the impact of drought on sugarcane development is of great interest. Plant growth-promoting bacteria hold the potential for improving tolerance to drought in agricultural systems. Thus, the present study aimed to evaluate whether inoculation with *Bacillus subtilis* can reduce the negative effects of drought on the nutritional, physiological, and morphological characteristics of sugarcane plants. For this, sugarcane was cultivated in a greenhouse, under controlled conditions of water and temperature, with the aid of four treatments: without and with inoculation of *B. subtilis*, in normal conditions of water availability, and in conditions of water restriction (2 × 2 factorial), with four replications. In treatments with inoculation, the pre-emerged seedlings were immersed in a *B. subtilis* solution and transplanted into experimental pots. Our results showed that inoculation with *B. subtilis* improved plant nutrition and chlorophyll concentrations. As a result, the gas exchange parameters (especially net photosynthetic rate and water use efficiency) were also improved, even under drought conditions. In addition, stress parameters (antioxidant metabolism activity) were reduced in inoculated plants. The sum of these beneficial effects resulted in increased root growth, tillering, stalk weight, and higher sucrose concentration in the stalks.

## 1. Introduction

Sugarcane (*Saccharum* spp.) is one of the most economically important crops worldwide due to its use as a raw material for sugar and biofuel production [1,2]. Sugarcane plants follow a semiperennial cycle of growth and are thus subject to periods of seasonal drought [3]. Climate change is increasing the frequency and severity of droughts, resulting in large sugarcane yield losses [4,5].

Water deficit is very complex, depending on the severity, duration, and phenological stage of the plant [6], which can affect its physiological, morphological, and biochemical characteristics [7]. In particular, water deficiency results in a decline in photosynthesis, with reduced leaf water potential and progressive reduction in CO_2_ assimilation rates, which are highly dependent on stomatal conductance [6,8,9]; reduces nutrient uptake; damages membranes [10]; and alters antioxidant metabolism in plants [11]. These changes induce the production of reactive oxygen species (ROS) and inactivation of antioxidant enzymes, resulting in an increase in cell damage [12]. These changes in antioxidant metabolism can reduce plant development and limit carbohydrate accumulation due to cellular damage [13,14].

Management strategies that optimize water use efficiency can mitigate the negative impacts of water stress on plant development and minimize damage. One potential strategy for improving tolerance to water deficit in agricultural production systems is the application of plant growth-promoting bacteria (PGPB) [15], which can aid in the acquisition of nutrients and synthesis of phytohormones, and promote improvement of the antioxidant system [16,17]. PGPR can improve plant performance by maintaining a level of ROS compatible with cellular functioning [18]. Among PGPB, *Bacillus* species secrete metabolites that stimulate plant growth and play important roles in biotic and abiotic stress tolerance [19]. In addition to helping plants resist pathogen attack [20,21], the *Bacillus subtilis* exerts stimulant and growth-protective effects on different plant species under various environmental stresses, including drought [22]. *B. subtilis* has great catabolic versatility, which favors plant growth under adverse conditions [23] through the synthesis of exopolysaccharides, siderophores, and plant hormones, and improvements in nutrient availability [19,24]. In addition, *B. subtilis* can increase the photosynthetic capacity of plants by influencing stomatal conductance and cellular tolerance to dehydration [25,26], promoting greater water use efficiency in plants [26].

Previous studies of *B. subtilis* have largely focused on its positive effects on short-cycle crops [17,26]. According to De lima et al. [17], the results obtained in their research with the inoculation of *B. subtilis* were beneficial in bean and corn plants, contributing to important photosynthetic characteristics under water stress. In addition, they found that the responses of each plant species were different to inoculation. Nevertheless, little is known about the impact of this microorganism on crops with longer cycles, such as sugarcane. For successful establishment, PGPB must not only interact favorably with the microbiota in the soil and environmental factors but also survive in the soil and be compatible with the crop [27]. The present study aimed to evaluate whether inoculation with *B. subtilis* can reduce the negative effects of drought on the nutritional, physiological and morphological characteristics of sugarcane plants. Here, we hypothesize that inoculation with *B. subtilis* in sugarcane can improve the physiological aspects and contribute to the better development of this crop under drought conditions. Crop nutritional parameters, photosynthetic pigment concentrations, gas exchange, antioxidant metabolism, and root-and-stalk growth were assessed in sugarcane plants that were subjected to water restriction (moderate drought stress) and uninoculated or inoculated with *B. subtilis* prior to seedling transplanting.

## 2. Materials and Methods

### 2.1. Plant Material and Growth Conditions

This experiment was performed under controlled conditions in a greenhouse located at São Paulo State University, Botucatu, Brazil (22°51′ S, 48°26′ W, 815 m asl). The greenhouse was maintained at a temperature between 22–32 °C, and 70% relative humidity. Sugarcane seedlings of variety RB855536 (RIDESA BRASIL, Araras, Brazil), which is considered sensitive to drought stress [28], were planted in polyethylene pots with a capacity of 38 dm^3^ of soil. Each pot was filled with 50 kg of red–yellow Latosol soil (soil bulk density: 1.43 g cm^3^). Soil acidity was corrected with the incorporation of lime in the proportion of 18 g 50 kg soil^−1^, to reach 70% base saturation, which was moistened for 30 days for acidity neutralization reactions to occur [29]. The soil of each pot was fertilized according to the needs discriminated in physicochemical analyses of the soil in the following amounts and sources [30,31,32]: 8.0 g of urea (45% of Nitrogen, N), 8.6 g of triple superphosphate (42% of P_2_O_5_ and 10% of Ca), 8.3 g of potassium chloride (60% of K_2_O), and 13.4 g of micronutrients containing 2% manganese (Mn), 1% copper (Cu), 10% zinc (Zn), and 0.2% molybdenum (Mo). The application of these fertilizers was based on the management practices used by sugarcane producers in Brazil.

### 2.2. Treatments and Experimental Design

The experiment was conducted in a 2 × 2 factorial scheme corresponding to the presence and absence of inoculation with *B. subtilis,* and the presence and absence of water restriction (moderate drought stress), respectively. The experimental design consisted of randomized blocks with four replicates. The biological inoculant was a commercial formulation of spores of *B. subtilis* UFPEDA 764 (Rizos OG^®^; minimum 3 × 10^9^ colony-forming units (CFU) mL^−1^, concentrated suspension; Lallemand, Patos de Minas, MG, Brazil). This product is registered with the Brazilian Ministry of Agriculture, Livestock and Food Supply. For the inoculated treatments, pregerminated seedlings were dipped in a solution of 1 × 10^10^ CFU mL^−1^ *B. subtilis* inoculant (UFPEDA 764) at 20 days after germination and then transplanted into the experimental pots.

For irrigation management, a soil water retention curve (SWRC) was established at a depth of 0.00–0.15 m. The daily water requirement was determined through readings obtained from a vacuum tensiometer, taken daily at 8:00–10:00 a.m. Based on the SWRC and data obtained from the tensiometer, daily amounts of irrigation were calculated using an electronic spreadsheet, aiming to raise soil moisture to field capacity (−10 kPa or 100% AWC = control treatment). Water deficit treatment was applied with the maintenance of soil moisture equal to 50% of field capacity. The water requirement was determined similarly to the previous treatment. Humidity at field capacity was maintained until the sugarcane plants reached the vegetative stage of tillering and canopy development [33]. Two top dressing fertilizations were carried out during this period: The first occurred 30 days after planting (DAP), with the application of 3.9 g of urea (45% N) and 4.3 g of potassium chloride (KCl, 60% K_2_O). The second topdressing occurred at 115 DAP with the application of 15 g of ammonium sulfate (21%, N, and 23% sulfur, S) and 2.8 g of KCl (60% K_2_O). Drought stress treatments started early in tillering and canopy development (~120 DAP).

### 2.3. Nutritional, Physiological and Biochemical Analyses

Approximately 60 days after the onset of drought stress, which corresponds to the grand growth phenological stage of sugarcane [33], sugarcane leaves (leaf +1) were collected between 9:00 a.m. and 10:00 a.m. to conduct nutritional, physiological and biochemical analyses.

#### 2.3.1. Sugarcane Crop Nutrition

Leaf macronutrient concentrations were determined from samples of dried and ground +1 leaves. The leaf samples were submitted to nitroperchloric acid digestion extraction. Then the content of phosphorus (P), potassium (K), calcium (Ca), magnesium (Mg), and sulfur (S) were determined by atomic absorption spectrophotometry. Leaf N content was determined by sulfuric acid extraction and quantified using the Kjeldahl distillation method, as suggested by the Association of Official Agricultural Chemists (AOAC) [34].

#### 2.3.2. Photosynthetic Pigments

To determine photosynthetic pigments, five discs were cut from fresh leaves between the margin and the leaf midvein. Each leaf disc was extracted in 2 mL of dimethylsulfoxide (DMSO) for 24 h in the dark at 25 °C. The absorbance was then read at wavelengths of 664, 647, and 480 nm, and the concentrations of chlorophyll *a*, chlorophyll *b,* and carotenoids, respectively, were calculated using the equations of Lichtenthaler [35].

#### 2.3.3. Gas Exchange

Sugarcane leaf gas exchange was evaluated using a portable infra-red gas analyzer CIRAS-3 (PP Systems, Amesbury, MA, USA), which was used to measure net photosynthesis (*A*; μmol CO_2_ m^−2^ s^−1^); stomatal conductance (*gs*; mol H_2_O m^−2^ s^−1^); substomatal CO_2_ concentration (*Ci*; mmol CO_2_ mol^−1^ air); leaf transpiration (*E*; mmol H_2_O m^−2^ s^−1^); water use efficiency [WUE; μmol CO_2_ (mmol H_2_O)^−1^]; and carboxylation efficiency, calculated from the ratio between *A*/*Ci*. Gas exchange analyses were performed between 8:00 a.m. and 10:00 a.m.

#### 2.3.4. Nitrate Reductase Activity

A 100 mg sample of fresh leaves was cut and transferred to assay tubes containing 3 mL of phosphate buffer solution (50 mM, pH 7.4) and 200 mM KNO_3_. The samples were vacuum infiltrated for 5 min and placed in a water bath at 33 °C for 30 min, protected from the light by using aluminum foil. Subsequently, 1 mL of 1% sulfanilamide was added to the 2M HCl solution. Then, 1 mL of 0.05% naphthylenediamine solution was added to stop the reaction as adapted from Reis et al. [36]. The absorbance was read in a spectrophotometer (Shimadzu uv 1800, Kyoto, Japan) at a wavelength of 540 nm and compared with a nitrite (NO_2_^−^) standard curve. The nitrate reductase (NR) activity was represented as nM NO_2_^−^ h^−1^ g FW^−1^.

#### 2.3.5. Preparation of Crude Extracts of Leaf Samples to Assess Antioxidant Metabolism

To obtain the crude extract, 0.3 g of leaf sample was ground under liquid nitrogen. Then, 5.0 mL of 0.1 M potassium phosphate buffer, pH 6.8, supplemented with 200 mg of polyvinylpolypyrrolidone (PVPP), were added to the sample [37]. Subsequently, the samples were subjected to centrifugation for 15 min at 5000 rpm, and the supernatant was used as the enzyme extract to determine total soluble protein and superoxide dismutase, catalase, and peroxidase activities.

#### 2.3.6. Total Soluble Protein Content

The determination of protein content occurred with the addition of a 50 mL aliquot of crude extract to 4950 μL of Bradford’s solution (Shimadzu uv 1800, Kyoto, Japan). Subsequently, spectrophotometric readings were performed at a wavelength of 595 nm. The total protein content was obtained using a standard curve made from bovine serum albumin [38]. From these results and the protein extract, superoxide dismutase (SOD), catalase (CAT), and peroxidase (POD) activities and proline content were determined.

#### 2.3.7. Superoxide Dismutase (SOD) and Peroxidase (POD) Activities

SOD activity (EC 1.15.1.1) was determined according to the method proposed by Giannopolitis et al. [37]. SOD activity was determined by monitoring the photochemical reduction of nitroblue tetrazolium (NBT) in a spectrophotometer (Shimadzu uv 1800, Kyoto, Japan) and readings carried out at a wavelength of 560 nm. SOD Activity is expressed as U SOD mg^−1^ protein.

POD activity (EC 1.11.1.7) was assayed according to the methodology of Peixoto et al. [38]. POD activity was determined by reading the absorbance at a wavelength of 420 nm of a solution formed by 50 mL of crude extract mixed with 4.95 mL of potassium phosphate buffer (25 mM, pH 6.8) containing 20 mM hydrogen peroxide (H_2_O_2_). The POD specific activity (mg protein^−1^) was calculated with a molar extinction coefficient of 2.47 mM^−1^ cm^−1^.

#### 2.3.8. Determination of Proline Content

To determine proline content, a mixture made with 2.0 mL of crude extract, 2.0 mL of acid ninhydrin, and 2.0 mL of glacial acetic acid was heated at 100 °C for 60 min. Subsequently, the absorbance reading was performed at a wavelength of 520 nm [39]. The determination of proline content was performed from a standard curve of pure proline, and the results were expressed in μmol g fresh weight^−1^ (FW).

#### 2.3.9. Sugar Concentrations

Reducing sugars, sucrose, and total soluble sugars were extracted and quantified according to the modified method of Xu et al. [40]. Ground leaf sample (0.1 g) was extracted with 80% (*v*/*v*) ethanol at 80 °C for 1 h, followed by centrifugation at 10,000 rpm for 15 min. Reducing sugars and sucrose were determined spectrophotometrically at wavelengths of 535 and 480, respectively, according to Somogyi–Nelson [41,42]. The ethanol-insoluble residue was used for starch extraction according to the protocols of Kuai et al. [43]. After removing ethanol by evaporation, 2 mL of deionized water was added, and the sample was incubated at 100 °C for 15 min. The starch was then hydrolyzed with 9.2 M and 4.6 M HClO_4,_ and determined spectrophotometrically using an anthrone reagent at a wavelength of 620 nm.

### 2.4. Root Parameters

Prior to the sugarcane harvest, root samples were removed using a probe with a diameter of 0.48 cm and a depth of 0.20 m. After being cleaned in running water, the samples were scanned in a digitizer coupled to a computer. The total length of the roots was determined by reading the images obtained from the scanner, using WinRhizo software version 3.8-b (Regent Instruments Inc., Quebec, QC, Canada) [44]. The length of the roots was determined considering the values obtained from the readings, converting them into m dm^−3^, from the volume of the pots and the probe used to collect the roots. Subsequently, the roots were subjected to drying in a forced-air oven at 60 °C until they reached constant weight. The dry weight of the roots was expressed in g dm^−3^.

### 2.5. Morphological Attributes

At harvest, the plant height (considering the height from the soil to the leaf +1), stalk diameter (measured at the internode of the first third from the ground), number of tillers, and leaf width (considering the middle third of the leaf +1) were measured. The sugarcane stalks were dried in an oven with forced-air circulation at 65 °C until reaching constant weight, in order to obtain the stalk dry matter.

### 2.6. Statistical Analysis

Data were first tested for normality through the Anderson–Darling test, and homoscedasticity was analyzed with Levene’s test. After meeting these prerequisites, the data were subjected to one-way analysis of variance (ANOVA) using the F test (*p* ≤ 0.05) and, when significant, the means were analyzed using Fisher’s protected least significant difference (LSD) test at *p* ≤ 0.05.

## 3. Results

### 3.1. Root Growth

**Figure 1:**
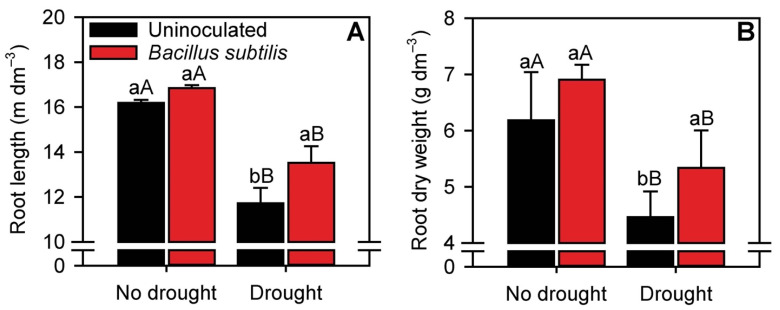
Root length (**A**) and root dry weight (**B**) of sugarcane plants in the different treatments. Columns with different capital letters indicate significant differences between the presence and absence of water restriction, and columns with different lowercase letters indicate significant differences between the presence and absence of *B. subtilis* inoculation, by Fisher’s protected LSD test at *p* ≤ 0.05. Error bars express the standard error of the mean (*n* = 4).

Sugarcane root length and root dry weight were not altered by inoculation with *B. subtilis* under normal water availability. By contrast, under moderate drought conditions, the root length and root dry weight of inoculated plants were approximately 20 and 22% higher than those of uninoculated plants, respectively (Figure 1A,B).

### 3.2. Sugarcane Crop Nutrition

**Figure 2:**
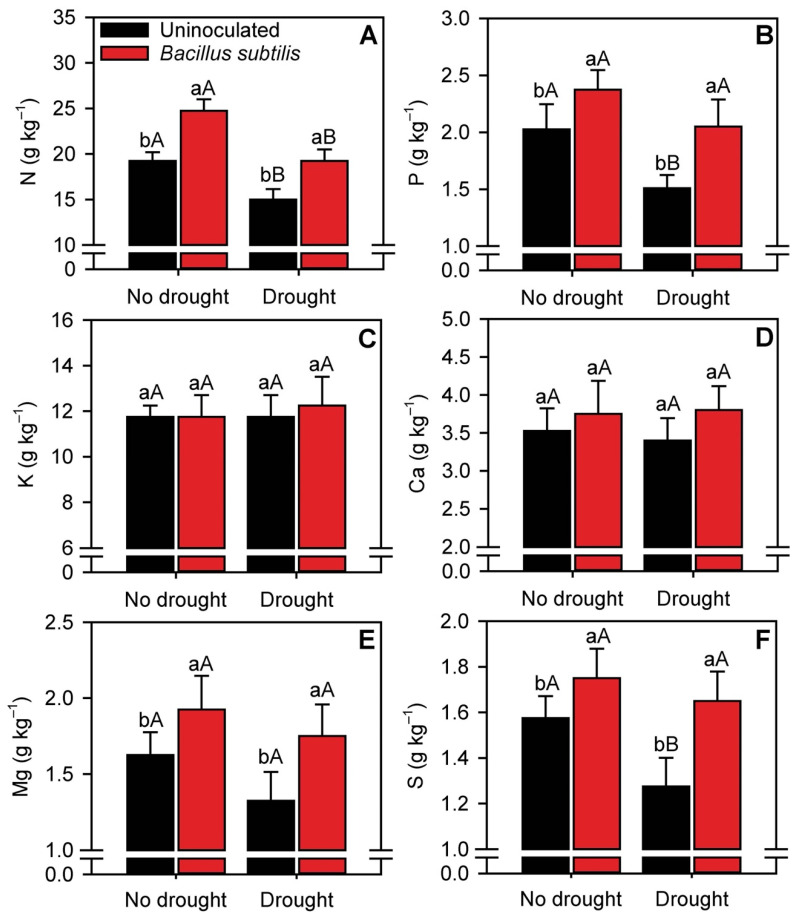
Concentrations of N (**A**), P (**B**), K (**C**), Ca (**D**), Mg (**E**), and S (**F**) in sugarcane leaves in the different treatments. Columns with different capital letters indicate significant differences between the presence and absence of water restriction, and columns with different lowercase letters indicate significant differences between the presence and absence of *B. subtilis* inoculation, by Fisher’s protected LSD test at *p* ≤ 0.05. Error bars express the standard error of the mean (*n* = 4).

*B. subtilis* inoculation significantly (*p* ≤ 0.05) increased leaf N, P, Mg, and S concentrations regardless of water availability (Figure 2A,B,E,F). Under normal water availability, N, P, Mg, and S concentrations were ~31.5, 20, 15, and 11% higher, respectively, in plants inoculated with *B. subtilis* than in uninoculated plants. In sugarcane plants established under moderate drought conditions, *B. subtilis* inoculation increased these same parameters by 20, 33, 28, and 29.5%, respectively. In general, drought reduced the concentrations of these nutrients, especially in uninoculated plants, whereas in inoculated plants, the concentrations were similar to those in plants established under normal water availability. In addition, the concentrations of K and Ca did not differ between treatments (Figure 2C,D).

### 3.3. Photosynthetic Pigments

**Figure 3:**
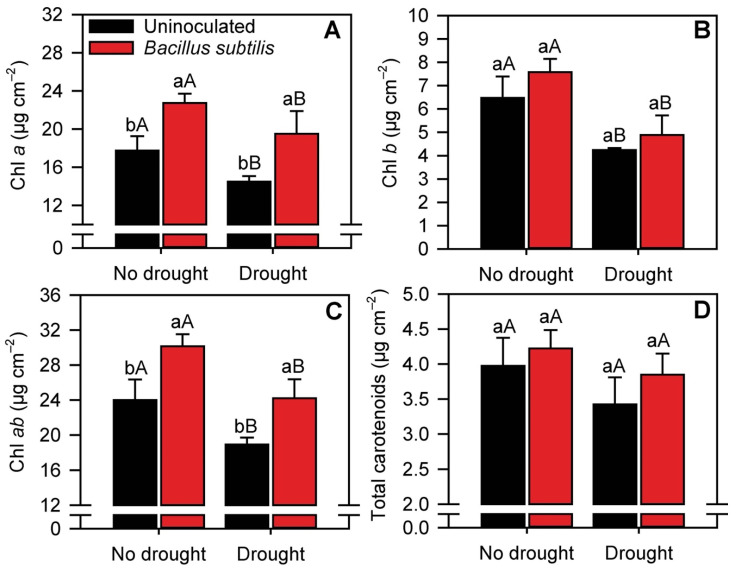
Concentrations of chlorophyll *a* (**A**), chlorophyll *b* (**B**), chlorophyll *ab* (**C**) and total carotenoids (**D**) in sugarcane leaves in the different treatments. Columns with different capital letters indicate significant differences between the presence and absence of water restriction, and columns with different lowercase letters indicate significant differences between the presence and absence of *B. subtilis* inoculation, by Fisher’s protected LSD test at *p* ≤ 0.05. Error bars express the standard error of the mean (*n* = 4).

The treatments significantly (*p* ≤ 0.05) affected the concentrations of all photosynthetic pigments (Figure 3A–C) except total carotenoids (Figure 3D). *B. subtilis* inoculation increased the concentrations of chlorophyll *a* (chl *a*) and total chlorophyll (chl *ab*) by 30 and 24% in sugarcane plants cultivated under normal water conditions and by 25 and 29% in drought-stressed plants. In addition, drought-stressed plants presented, on an average, ~35% lower chlorophyll concentrations than nonstressed plants. The concentration of chlorophyll *b* was influenced only by drought, which reduced its concentration by 55% (considering the average concentration in uninoculated and inoculated plants) (Figure 3B).

### 3.4. Leaf Gas Exchange

**Figure 4:**
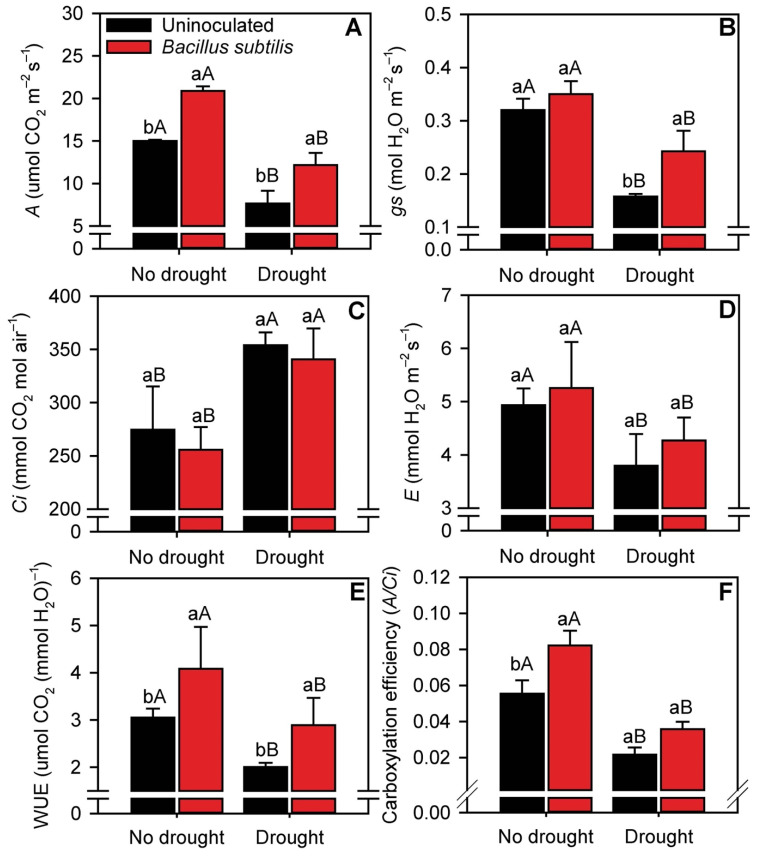
Net photosynthetic rate (*A*) (**A**), stomatal conductance (*gs*) (**B**), internal CO_2_ concentration (*Ci*) (**C**), leaf evapotranspiration (*E*) (**D**), water use efficiency (*WUE*) (**E**), and carboxylation efficiency (**F**) in sugarcane leaves in the different treatments. Columns with different capital letters indicate significant differences between the presence and absence of water restriction, and columns with different lowercase letters indicate significant differences between the presence and absence of *B. subtilis* inoculation, by Fisher’s protected LSD test at *p* ≤ 0.05. Error bars express the standard error of the mean (*n* = 4).

*B. subtilis* inoculation increased (*p* ≤ 0.05) the net photosynthetic rate (*A*) by 40 and 67% in sugarcane plants grown under normal water availability and drought conditions, respectively (Figure 4A). *B. subtilis* inoculation affected stomatal conductance (*gs*) only in drought-stressed plants, in which *gs* was 37% higher than in uninoculated plants (Figure 4B). On an average, drought decreased *A* and *gs* values by ~39% compared with nonstressed plants. The substomatal CO_2_ concentration (*Ci*) increased by 31% and leaf transpiration (*E*) decreased by 20% in drought-stressed plants, regardless of *B. subtilis* inoculation (Figure 4C,D). Interestingly, water use efficiency (WUE) and carboxylation efficiency were influenced by the combination of *B. subtilis* inoculation and water availability (Figure 4E,F). *B. subtilis* inoculation increased the WUE of sugarcane plants by 32 and 40% under normal water availability and drought conditions, respectively, and the carboxylation efficiency was 57% higher in nonstressed plants.

### 3.5. Carbohydrate Metabolism

**Figure 5:**
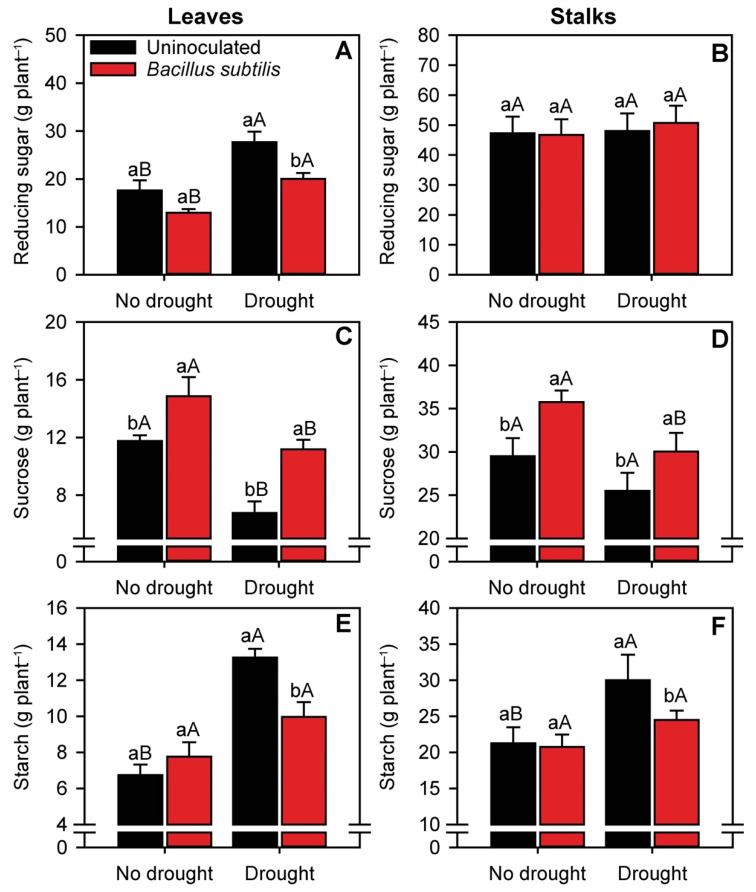
Concentrations of reducing sugars (**A**,**B**), sucrose (**C**,**D**), and starch (**E**,**F**) in sugarcane leaves and stalks, respectively, in the different treatments. Columns with different capital letters indicate significant differences between the presence and absence of water restriction, and columns with different lowercase letters indicate significant differences between the presence and absence of *B. subtilis* inoculation, by Fisher’s protected LSD test at *p* ≤ 0.05. Error bars express the standard error of the mean (*n* = 4).

The concentrations of carbohydrates were determined in both sugarcane leaves and stalks (Figure 5). The concentrations of reducing sugars (leaves) and starch (leaves and stalks) were significantly higher (*p* ≤ 0.05) under water deficit, especially in the absence of *B. subtilis* inoculation (Figure 5A,B,E,F). On an average, in drought-stressed plants, *B. subtilis* inoculation decreased the leaf concentrations of reducing sugars by 30% and the leaf and stalk concentrations of starch by 26 and 20%, respectively. Conversely, *B. subtilis* inoculation increased leaf sucrose concentrations by 25% in nonstressed plants and by 52% in drought-stressed plants (Figure 5C). Similarly, *B. subtilis* inoculation increased the stalk sucrose concentration by 27% in nonstressed plants and 20% in drought-stressed plants (Figure 5D).

### 3.6. Enzymatic Activity and Proline Content

**Figure 6:**
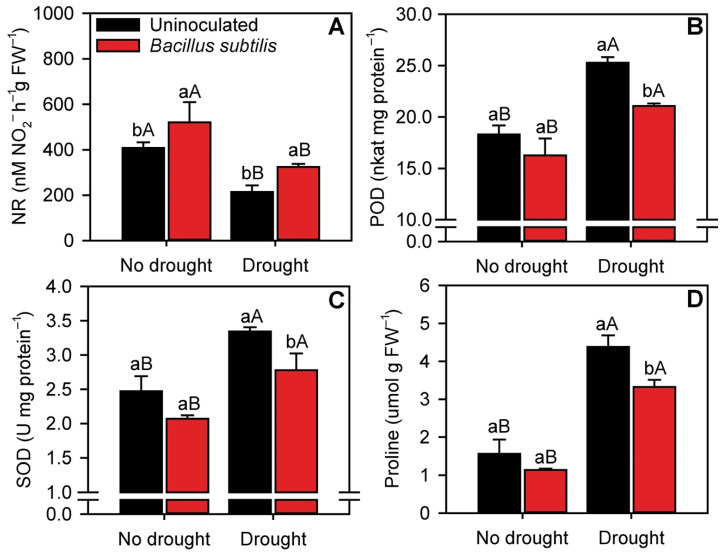
Activities of nitrate reductase (NR) (**A**), peroxidase (POD) (**B**), and superoxide dismutase (SOD) (**C**) and proline (**D**) content in sugarcane leaves in the different treatments. Columns with different capital letters indicate significant differences between the presence and absence of water restriction, and columns with different lowercase letters indicate significant differences between the presence and absence of *B. subtilis* inoculation, by Fisher’s protected LSD test at *p* ≤ 0.05. Error bars express the standard error of the mean (*n* = 4).

*B. subtilis* inoculation significantly increased (*p* ≤ 0.05) NR activity by 28 and 55% in nonstressed and drought-stressed plants, respectively (Figure 6A). Overall, drought reduced NR activity by 56%, considering the average between inoculated and uninoculated plants. The activities of the antioxidant enzymes POD and SOD increased in drought-stressed plants (Figure 6B,C). Under normal water availability, *B. subtilis* inoculation did not influence POD and SOD activities, but under drought conditions, inoculation decreased POD activity by 19% and SOD activity by 17%. Furthermore, *B. subtilis* inoculation reduced proline content by 25% in drought-stressed plants but had no impact on proline content in nonstressed plants (Figure 6D).

### 3.7. Sugarcane Biometric Parameters

**Figure 7:**
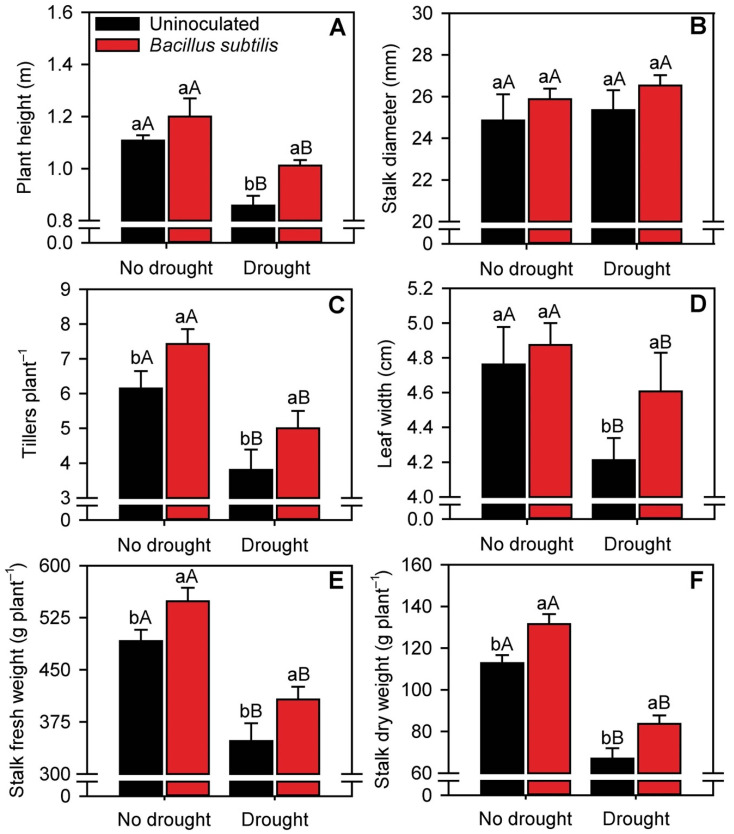
Plant height (**A**), stalk diameter (**B**), number of tillers per plant (**C**), leaf width (**D**), stalk fresh weight (**E**), and stalk dry weight (**F**) of sugarcane plants in the different treatments. Columns with different capital letters indicate significant differences between the presence and absence of water restriction, and columns with different lowercase letters indicate significant differences between the presence and absence of *B. subtilis* inoculation, by Fisher’s protected LSD test at *p* ≤ 0.05. Error bars express the standard error of the mean (*n* = 4).

Drought stress significantly decreased (*p* ≤ 0.05) plant height, and *B. subtilis* inoculation increased plant height by 40% compared with uninoculated plants (Figure 7A). Stalk diameter was not altered by the treatments (Figure 7B). Interestingly, *B. subtilis* inoculation increased plant tillering in both nonstressed (23%) and drought-stressed (29%) plants (Figure 7C). In addition, drought reduced the average number of tillers per plant by 34%. *B. subtilis* increased leaf width by 9.5% in drought-stressed plants. Stalk fresh and dry weight per plant decreased by 20 and 56%, respectively, under drought conditions, considering the average between uninoculated and inoculated plants (Figure 7E,F). Furthermore, *B. subtilis* inoculation increased stalk fresh weight by 12% in nonstressed plants and by 18% in drought-stressed plants. Finally, inoculation increased stalk dry weight regardless of water availability (normal water availability: 18%; drought: 26%).

## 4. Discussion

Many microbes produce substances that enhance plant health and growth [45,46]. Among PGPB, *B. subtilis* plays a significant role in phytohormone production and biocontrol via the induction of systemic resistance [47]. Most studies of *B. subtilis* in plants have focused on controlling and preventing plant pathogen infection [19,48] and determining how bacterial metabolites act as biologically active substances (antimicrobials and antibiotics) that protect the plant [49,50]. Efforts to characterize the plant growth-promoting effects of this bacterium and its contribution to minimizing abiotic stresses such as drought have been limited, and generated conflicting results. Water deficit has severe effects on the development of plants, compromising their cellular, metabolic, and physiological activities, and consequently, there is a reduction in productivity [51,52]. Our findings provide insights into the physiological mechanisms, i.e., carbohydrate metabolism and antioxidant metabolism, by which *B. subtilis* minimizes the effects of water deficit and promotes sugarcane plant development.

*B. subtilis* inoculation improved several growth parameters, including root length and biomass, under conditions of low water availability. These changes in turn facilitated the assimilation of nutrients under adverse conditions, as evidenced by the increases in leaf concentrations of nutrients in sugarcane inoculated with *B. subtilis* (Figure 2). The improvements in the root system may have been due to metabolites produced by *B. subtilis*, which can increase stress tolerance in the plant host and induce the expression of stress response genes, phytohormones, and related metabolites [47].

Plant nutrition and development are strictly influenced by drought [52,53], and this condition reduces the efflux of macro and micronutrients [54,55]. However, *B. subtilis* inoculation increased leaf concentrations of N, P, Mg, and S, even under water restriction. The increase in leaf N concentrations in inoculated plants may have been due to higher NR activity. Interestingly, leaf S concentrations were also higher in inoculated plants. There is evidence of synergism in the uptake of N and S by plants [56], and increased NR activity has been correlated with increased S levels, reinforcing the role of S in N metabolism [57,58]. There is also synergism of P and Mg uptake by plants [59]. We observed an increase in Mg in plants inoculated with *B. subtilis*, which increases soil P solubilization via mechanisms [47] such as acidification, chelation, and production of organic acids [60]. These mechanisms contribute to the improved use of soil and fertilizer P by plants. Thus, the increase in Mg concentrations in inoculated plants may reflect the effects of *B. subtilis* on P solubilization and uptake.

Under moderate drought conditions, photosynthetic pigments were reduced in the present study, possibly due to degradation by ROS [61]. Water restriction causes a cellular redox imbalance due to increased production and accumulation of ROS, which interferes with metabolic processes [62]. This increase exceeds the ability of antioxidant enzymes to maintain cellular balance, resulting in a negative balance between ROS production and elimination [63,64], and can lead the photooxidation of pigments and chlorophyll degradation [53]. These effects negatively impact plant development, as chlorophyll levels are directly linked to photosynthetic capacity and plant growth [6,65]. N and Mg concentrations were low in drought-stressed plants, consistent with the structural roles of these elements in chlorophyll [66]. However, *B. subtilis* inoculation decreased chlorophyll degradation induced by water restriction, possibly by mitigating the effects of drought stress on the plant and increasing the activities of antioxidant enzymes [47]. Increased chlorophyll content is essential for maintaining normal photosynthesis under stress [67]. The inoculated plants had increased chlorophyll levels even under normal conditions of water availability.

Consistent with the increase in leaf chlorophyll concentrations, gas exchange parameters were improved in plants inoculated with *B. subtilis*. Even under water restriction, *B. subtilis* inoculation increased *A* and *gs* values in sugarcane plants. Drought stress can reduce photosynthetic activity by causing turgor pressure dysfunction, stomatal closure (faster process induced by drought) [68], low gas exchange, and low CO_2_ assimilation, leading to impairment of the photosynthetic apparatus [69,70]. The improvement in stomatal conductance in drought-stressed plants inoculated with *B. subtilis* may have been a crucial factor for the maintenance of photosynthetic activity, since stomatal conductance is one of the factors that most affect the efficiency of photosynthesis under drought conditions [71,72]. This is because stomatal closure reduces the efficiency of CO_2_ assimilation and increases metabolic reactions that cause photodamage [53,73]. The inoculated plants also presented higher carboxylation efficiency as a result of the increased net photosynthetic rate and use of substomatal CO_2_. The greater leaf width of inoculated plants may be another factor that contributed to the increase in the net photosynthetic rate. Even under water deficit, inoculation with *B. subtilis* increased the photosynthetically active area of the leaf blade. Limiting leaf growth is among the first visible impacts of water stress because leaves determine radiation interception and are the main photosynthetic organs [74]. This limitation of leaf growth occurs in order to achieve a balance between the water absorbed by the plant roots and the water status of the plant tissues [6,75]. Water restriction reduces the leaf area and, consequently, carbohydrate metabolism [76]. The greater plant levels of N, P, Mg, and S, which are linked to the ability of *B. subtilis* to stimulate plant hormone production [77], may have contributed to an increase in leaf blade area.

The impairment of photosynthesis by drought can lead to ROS formation via misdirection of electrons in the photosystems [77]. Decreasing the fixation of CO_2_ by photosynthesis increases the accumulation of electrons in photosystems I and II, resulting in greater ROS generation and lipid peroxidation [78]. Although ROS levels were not measured in this study, the higher proline content in drought-stressed plants confirms this hypothesis. Proline acts as a major N and energy reservoir for utilization upon exposure to stresses such as drought [49]. The lower proline content in inoculated plants, compared with uninoculated plants, indicates a lower level of stress. In addition, the activities of the ROS-scavenging enzymes SOD and POD increased in drought-stressed plants [79]. ROS scavenging helps plants resist drought [52,80] and maintain normal metabolic processes [81]. Interestingly, the activities of these enzymes were reduced in inoculated sugarcane plants, possibly due to the lower levels of stress compared with uninoculated plants. ROS production is higher in stressed plants, which leads to increases in the activities of antioxidant enzymes that use ROS as a reaction substrate [82]. Numerous studies have demonstrated that inoculation with PGPB such as *B. subtilis* is a feasible strategy for reducing ROS concentrations in the tissues of drought-stressed plants [70,78,83].

Sugarcane plants inoculated with *B. subtilis* exhibited higher production of sucrose in the leaves, culminating in greater transport of this sugar to the stalk, even under drought conditions. Sucrose is the primary product of photosynthetic tissues and the main sugar transported from source to sink tissues via phloem [84]. The stalks are the final destination of most of the sucrose produced in sugarcane. Unlike sucrose, leaf concentrations of reducing sugars (fructose + glucose) increased under drought conditions and were even higher in uninoculated plants. By contrast, the stalk concentrations of reducing sugars were not affected by the treatments. Under drought stress, leaf sucrose is inverted into reducing sugars by the enzymes sucrose synthase and invertase [85]. Starch also increases in drought-stressed plants [86,87]. In general, under stressful environmental conditions, reducing sugars act as essential metabolites to keep plant metabolism active and maintain an adequate energy supply [86]. The concentration of starch also increases, as starch is stored in chloroplasts to serve as an energy reservoir when photosynthesis is limited [87]. In summary, under drought conditions, *B. subtilis* inoculation efficiently increased photosynthesis, reduced stress levels, and promoted the production and accumulation of sucrose in sugarcane stalks. In the absence of inoculation, plants grown under drought stress had higher levels of reducing sugars and starch, which drastically reduces the quality of sugarcane as a raw material for industrial use [88].

Finally, the biometric parameters of the uninoculated sugarcane plants were strongly affected by water restriction. Plant height and the fresh and dry weight of stalks were significantly lower in drought-stressed and uninoculated plants. Water stress greatly impairs the physiological and biochemical processes of plants, and limits the absorption of nutrients from the soil solution by roots for distribution to the shoots [53]. These factors negatively affect plant growth and development, and biomass accumulation, culminating in low yields [82]. Thus, inoculation of plants with *B. subtilis* improved nutrition, gas exchange and antioxidant metabolism in sugarcane plants under drought stress, resulting in significant increases in sucrose content and stalk production (fresh and dry weight).

## 5. Conclusions

Here we reported how *Bacillus subtilis* can improve the tolerance of sugarcane plants to water stress. Our results showed that the inoculation of sugarcane seedlings with *B. subtilis* altered the nutritional, physiological and growth parameters of both nonstressed and drought-stressed plants. In sugarcane plants established under drought stress, *B. subtilis* inoculation increased N, P, Mg, and S concentrations in the leaves, chlorophyll concentration, net photosynthetic rate, and promoted greater water use efficiency. We also observed decreases in parameters related to stress levels (SOD and POD activities and proline concentration). These cascading effects potentiated root development, tillering, stalk weight, and stalk sucrose concentration, demonstrating that *B. subtilis* inoculation is an important tool to reduce the negative effects of drought stress in sugarcane. These results have important implications for farmers, as sugarcane fields are continually subject to periods of drought during the crop cycle. The findings indicate that *B. subtilis* inoculation is a viable option for improving the physiological parameters of sugarcane and mitigating the negative effects of water restriction. Our results provide a comprehensive survey of the response of *B. subtilis* in the sugarcane crop, and new insights to improve the sustainability of sugarcane fields in tropical regions, environments with predisposition to dry periods and that which strongly reduce the productive capacity of this energy crop. However, our study was conducted under controlled greenhouse conditions, and further studies under field conditions are needed to validate these effects, including the impact of additional applications during the crop cycle and the effect of inoculation on subsequent sugarcane ratoons.

## Data Availability

The datasets analyzed during the current study are available from the corresponding author upon reasonable request.
